# A Pilot Experiment to Measure the Initial Mechanical Stability of the Femoral Head Implant in a Cadaveric Model of Osteonecrosis of Femoral Head Involving up to 50% of the Remaining Femoral Head

**DOI:** 10.3390/medicina59030508

**Published:** 2023-03-05

**Authors:** Seungha Woo, Youngho Lee, Doohoon Sun

**Affiliations:** Department of Orthopedic Surgery, Daejeon Sun Hospital, 29 Mokjung-ro, Jung-gu, Daejeon 34811, Republic of Korea

**Keywords:** bone defect, femoral head, osteonecrosis of femoral head, resurfacing arthroplasty, stability

## Abstract

*Background and Objectives:* Currently, only patients with osteonecrosis of the femoral head (ONFH), who had bone defects involving 30–33.3% of the remaining femoral head, are indicated in hip resurfacing arthroplasty (HRA). In an experimental cadaver model of ONFH involving up to 50% of the remaining femoral head, the initial stability of the femoral head implant (FHI) at the interface between the implant and the remaining femoral head was measured. *Materials and Methods:* The ten specimens and the remaining ten served as the experimental group and the control group, respectively. We examined the degree of the displacement of the FHI, the bonding strength between the FHI and the retained bone and that at the interface between the FHI and bone cement. *Results:* Changes in the degree of displacement at the final phase from the initial phase were calculated as 0.089 ± 0.036 mm in the experimental group and 0.083 ± 0.056 mm in the control group. However, this difference reached no statistical significance (*p* = 0.7789). Overall, there was an increase in the degree of displacement due to the loading stress, with increased loading cycles in both groups. In cycles of up to 6000 times, there was a steep increase. After cycles of 8000 times, however, there was a gradual increase. Moreover, in cycles of up to 8000 times, there was an increase in the difference in the degree of displacement due to the loading stress between the two groups. After cycles of 8000 times, however, such difference remained almost unchanged. *Conclusions:* In conclusion, orthopedic surgeons could consider performing the HRA in patients with ONFH where the bone defects involved up to 50% of the remaining femoral head, without involving the femoral head–neck junction in the anterior and superior area of the femoral head. However, more evidence-based studies are warranted to justify our results.

## 1. Introduction

Osteonecrosis of the femoral head (ONFH), also referred to as avascular necrosis, is defined as a pathologic condition arising from an ischemic injury that is characterized by both a crucial disruption of blood supply to the bone and an increase in the intraosseous pressure. Subsequently, this results in the degradation of the organic elements of the bone and the marrow, thus commonly leading to a collapse of subchondral bone in the femoral head [1,2,3,4]. As such, ONFH is a debilitating, progressive joint disease of idiopathic origin; it is an interesting topic from both clinical and economic perspectives [5,6,7]. Over the past few years, there has been an increase in the prevalence of ONFH [8]. Moreover, it has been diagnosed with increasing frequency in young adults and has a significant socioeconomic impact [9]. The annual number of patients who are hospitalized for the treatment of ONFH is estimated at 10,000–20,000 in the US [5]. It is a serious disease entity that may affect the quality of life in patients with ONFH [10]. Still, however, little is known about the risk factors associated with its pathogenesis and pathophysiology, although they include the long-term use of chronic steroids, smoking, alcoholism, hip trauma and prior hip surgery [11,12]. This makes it difficult to define surgical methods and curative effects [1,13]. It is, therefore, crucial to obtain a better understanding of the pathogenesis of and make therapeutic approaches to ONFH [8]. Despite recent advancements in diagnostic modalities, effective treatments have been elusive and a majority of cases of ONFH eventually result in a collapse of the femoral head. Most of the surgical modalities for patients with ONFH aim to prevent the collapse of the subchondral bone, although their clinical outcomes have been reported to be inconsistent [1,13]. Core decompression may be effective for the treatment of early-stage ONFH, although femoral osteotomy, vascularized or non-vascularized bone grafting and total hip arthroplasty (THA) may also be attempted for that of advanced ONFH [14].

Patients with ONFH account for 5–12% of those undergoing THA in the US [1]. If treated conservatively, >80% of affected hips would progress to femoral collapse and the destruction of the hip joint within four years of initial diagnosis; this often requires THA [15]. In the early stage of ONFH, joint-preserving surgical techniques are often considered. However, this causes problems, such as a significant failure rate and morbidity [16,17,18,19]. THA is often a mainstay of treatment in patients with osteonecrosis of the hip [18]. However, it may be not an attractive treatment option for younger patients; it would be desirable to avoid or delay THA. This is not only because most of the younger patients would outlive the current state-of-the art implants, but also because it has been suggested that such patients are less satisfied with its clinical outcomes [17,20]. It is therefore imperative that effective treatment modalities be developed, which would be essential for preventing the collapse of affected femoral heads or prolonging the interval between initial diagnosis and THA [7]. To date, diverse small animal models using rats or rabbits have been used to develop new treatment modalities for ONFH. Thus, these animal models induce ONFH by systematic insult, including steroid administration or steroid combined with another adjunct agent [21,22,23,24,25,26,27]. It would also be mandatory, however, to improve the relevance of animal models of ONFH in a clinical setting.

Both hemi-resurfacing arthroplasty and metal-on-metal hip resurfacing arthroplasty (HRA) are alternatives to conventional THA for patients with ONFH [28,29,30]. Hemi-resurfacing and total resurfacing arthroplasty are referred to as the prosthetic replacement of the femoral side only and that of both the femoral head and the acetabular surface, respectively [31]. According to a US nationwide study, the frequency of THA was the highest (90%), followed by HRA (0.2%) and osteotomy (1%) [32]. Both hemi-resurfacing arthroplasty and HRA are potentially advantageous in preserving bone and the loading of the proximal femur, lowering a risk of dislocation and eliminating the polyethylene debris that may cause osteolysis as compared with conventional THA [30,33,34]. Consequently, HRA is considered an appropriate option for young and active patients with ONFH [35].

However, there are things to consider regarding the indications of HRA in the context of regulatory requirements enforced by the US Food and Drug Administration (FDA). In 2006, the US FDA approved the clinical use of a metal-on-metal (MoM) resurfacing implant for primary HRA. This is based on the pre-market approval (PMA) process in 2385 patients with non-inflammatory or inflammatory arthritis receiving the Birmingham Hip Resurfacing (BHR) System (Smith & Nephew Orthopaedics, Memphis, TN) [36,37]. Later, in 2007 and 2009, the US FDA approved the clinical use of two additional MoM resurfacing implants, such as the Cormet™ Hip Resurfacing System (Corin, Tampa, FL, USA) and the Conserve^®^ Plus Total Hip Resurfacing System (MicroPort Orthopedics, Boston, MA, USA), respectively, in patients with non-inflammatory degenerative or inflammatory arthritis [38,39]. Since then, the US FDA has cleared a variety of implants for marketing through the 510(k) process [40]. According to the US FDA, however, patients with ONFH who had a necrotic area involving >50% of the femoral head are contraindicated in the use of MoM implants [41].

Given the above background, we created an experimental cadaver model of ONFH involving 50% of the remaining femoral head. We conducted this study to measure the initial stability of the FHI at the interface between the implant and the remaining femoral head.

## 2. Materials and Methods

### 2.1. Experimental Materials and Setting

We conducted the current biomechanical study using ten pairs of specimens from ten cadavers (*n* = 10). The specimens were preserved in a frozen state (−20 °C), and were gradually defrosted at room temperature for 24 h. A total of 20 specimens were equally divided into the experimental group (*n* = 10) and the control group (*n* = 10).

Inclusion criteria for the current experiment were a lack of pathologic bone lesions and the Singh index > 5. The Singh index is a typical classification system for the bone density of the femoral neck based on the qualitative visibility of the trabecular patterns [42].

**Figure 1:**
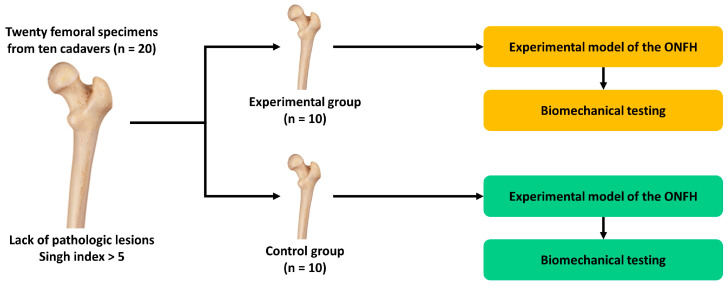
Experimental schema. Abbreviation: ONFH, osteonecrosis of femoral head.

The experimental procedures are schematically shown in Figure 1.

### 2.2. Creation of an Experimental Model of ONFH

**Figure 2:**
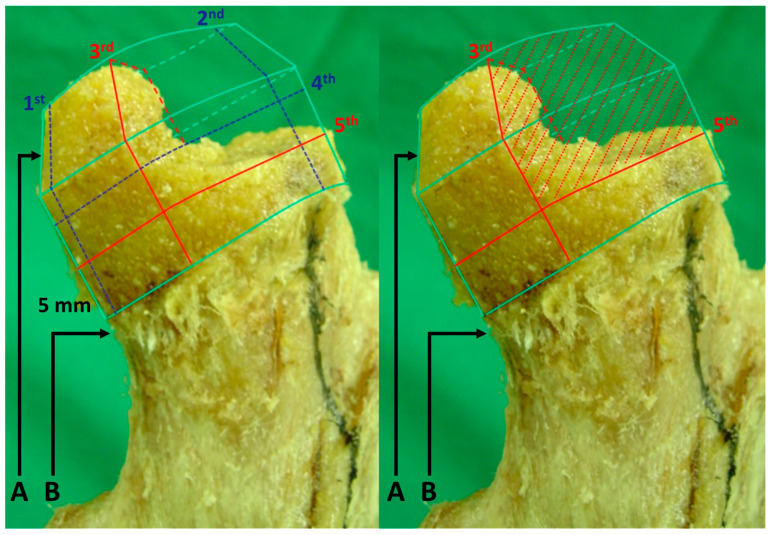
Anatomic specimens of the femur obtained from ten cadavers. Note: A: Femoral head, B: Femoral neck. We drew the 1st and 2nd lines along the midline of the femoral neck on the anterior–posterior and lateral plane, respectively. Then, we drew the 3rd line that crosses the 1st and 2nd lines from postero-superior to infero-anterior directions. We also drew the 4th line, that was vertical to the 3rd line and then crossed the center of the femoral head. In parallel with the 4th line, we drew the 5th line at 5 mm proximal to the head–neck junction. Finally, we dissected 50% of the antero-superior part of the remaining femoral head based on the 3rd and 5th lines.

With a free-hand technique at an angle of 135° to the axis of the femoral shaft on the anterior–posterior plane and in parallel with the central axis of the femoral neck, we placed the femoral guide pin on the lateral plane. This was followed by the femoral reaming using a cannulated sleeve and a chamfering reamer with an appropriate size. After saline irrigation, we confirmed a lack of notching and inappropriate exposure of the cancellous bone at the femoral head–neck junction. Then, we dissected 50% of the antero-superior area of the remaining femoral head, and thereby caused bone defects in the experimental group. To consistently make bone defects, we mapped the area in dissecting the area of the femoral head (Figure 2).

Then, we placed an MoM implant (Durom^®^; Zimmer Inc., Warsaw, IN, USA) in the bone defect area and then fixed it using low-viscosity bone cement (Surgical Simplex^®^ P; Stryker Howmedica Osteonics Corp., Rutherford, NJ, USA) for both groups. In the experimental group, however, we performed the same maneuver after restoring the bone defect area using a sufficient amount of low-viscosity bone cement.

### 2.3. Assessment of the Biomechanical Stability of the Specimen

**Figure 3:**
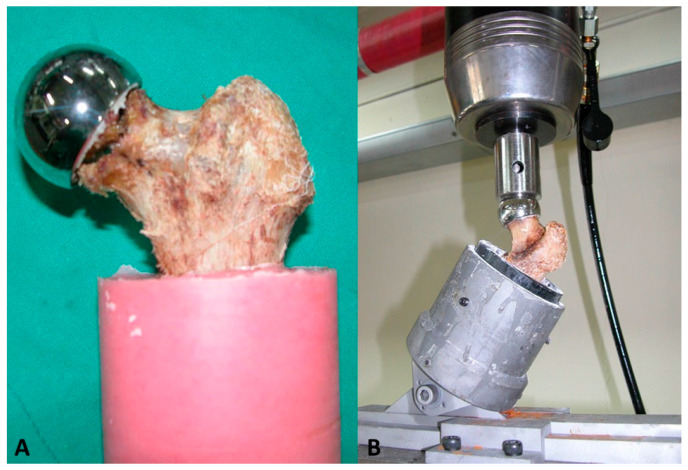
The preparation of the femoral specimens. (**A**) The femoral specimen was inserted in the resin block along the anatomical axis. (**B**) The femoral specimen with the femoral head implant was placed in a custom-made jig for the loading–unloading test.

We transected the specimen at the isthmus and then fixed it using a resin fixative (Vertex Self-Curing; Vertex-Dental B.V., Soesterberg, The Netherlands) (Figure 3A). We placed it on the machine at a valgus angle of 30°, thus attempting to preventing the fracture of the femoral neck while repeatedly applying a mechanical load to it.

We measured the biomechanical stability of the specimen using the dynamic testing machine based on the biaxial fluid pressure (Instron 8500^®^; Instron Corp., Norwood, MA, USA), for which we repeatedly applied a loading stress at a constant rate of 2 Hz [43,44]. The magnitude of loading stress ranged between 60 and 300 kg; it was five times higher as compared with the non-loading condition. The loading and displacement were measured at a sampling rate of 20 Hz using MAX^TM^ software (Instron Corp.) in a total of 15,000 cycles (Figure 3B) [45]. Then, we measured the strength against the displacement of the FHI due to the loading stress [46,47]. In each cycle of loading, we measured the degree of the displacement of the FHI through a scatter plotting analysis using a load versus displacement graph [48,49]. In measuring the bond strength, we defined the initial and final phase of loading as that applied to the specimen in cycles ranging from 1 to 5000 times and 10,000 to 15,000 times, respectively. Thus, we compared differences in changes in the bond strength at the final phase from the initial phase between the two groups, for which we maintained the degree of loading stress consistently throughout the experiment. Therefore, the magnitude of bond strength was solely dependent on the degree of the displacement of the FHI.

### 2.4. Scanning Electron Microscopy (SEM)

**Figure 4:**
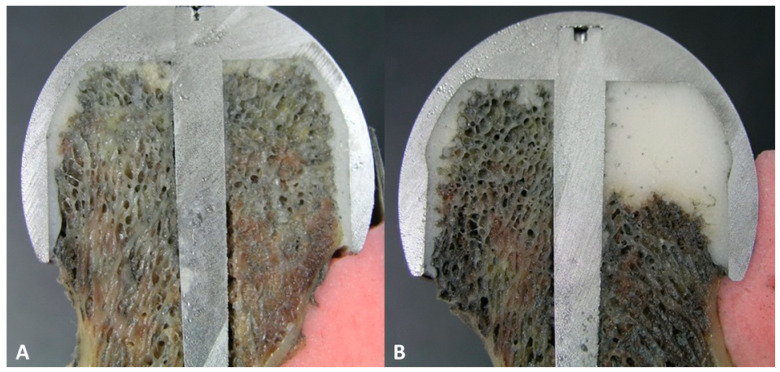
Cross sections of the femoral specimens. (**A**) In the control group, bone cements were used to fill the gap between bone and the implant. There were no other bone defects filled with bone cement. (**B**) In the experimental group, bone defects were used to sufficiently fill 50% of the bone defects. There were no other bone defects.

After selecting four pairs of the specimen obtained from the same cadavers in both groups, we prepared cross-sectional samples by pulling the diamond saw across the center of the bone defects on the coronal plane. Thus, we attempted to measure the bond strength at the largest bone defects (Figure 4).

The samples were completely frozen in a refrigerator (−80 °C) for 24 h and then underwent a freeze-drying process at a temperature of −77 °C for two days. We therefore prepared dry femoral samples. This was followed by SEM to examine the bond strength both at the bone–cement interface and at the implant–cement interface.

### 2.5. Statistical Analysis

Statistical analysis was carried out using the SPSS version 25.0 (IBM Corp., Armonk, NY, USA). All data were presented as mean ± SD (SD: standard deviation). We compared differences in the size of the FHI and changes in the bond strength at the final phase from the initial phase between the two groups using the Student’s *t*-test. A *p*-value of <0.05 was considered statistically significant.

## 3. Results

### 3.1. Size of the FHI

The mean size of the FHI was 49.4 ± 2.1 (range, 44–53) mm in the experimental group and 49.1 ± 1.8 (range, 43–52) mm in the control group. However, this difference reached no statistical significance (*p* = 0.7356).

### 3.2. Results of the Biomechanical Study

**Figure 5:**
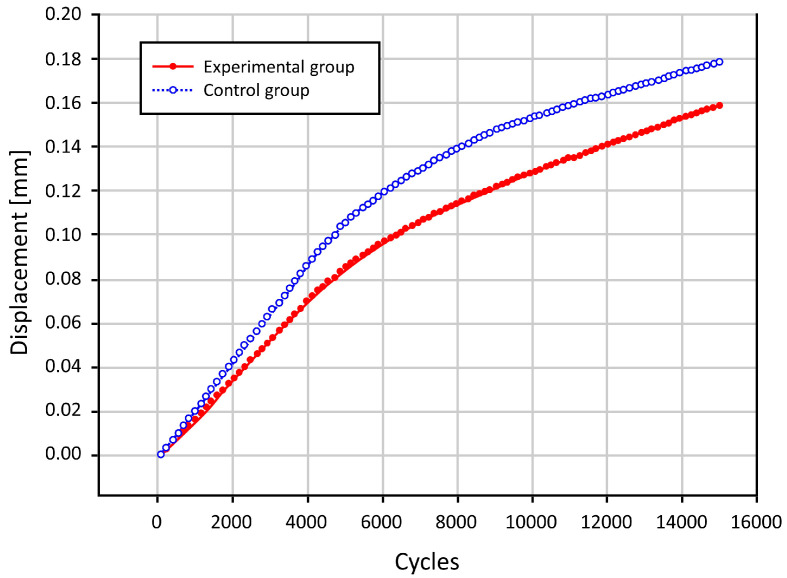
The degree of the displacement of the femoral head implant.

Overall, there was an increase in the degree of displacement due to the loading stress with increased loading cycles in both groups. In cycles of up to 6000 times, there was a steep increase. After cycles of 8000 times, however, there was a gradual increase. Moreover, in cycles of up to 8000 times, there was an increase in the difference in the degree of displacement due to the loading stress between the two groups. After cycles of 8000 times, however, such difference remained almost unchanged (Figure 5).

**Figure 6:**
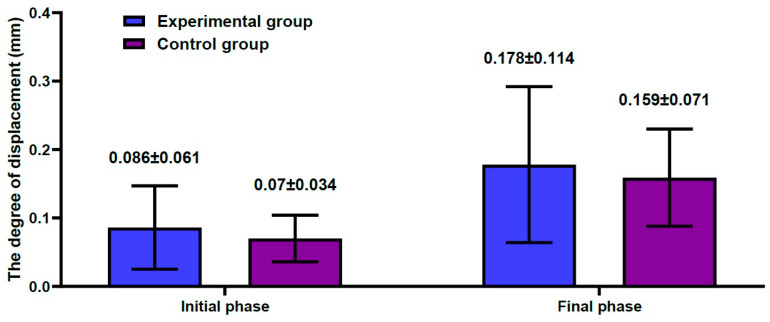
Changes in the degree of displacement at the final phase from the initial phase.

The degree of displacement at each phase is represented in Table 1. Changes in the degree of displacement at the final phase from the initial phase were calculated as 0.089 ± 0.036 mm in the experimental group and 0.083 ± 0.056 mm in the control group. However, this difference reached no statistical significance (*p* = 0.7789) (Figure 6).

### 3.3. The Bond Strength at the Bone–Cement Interface

**Figure 7:**
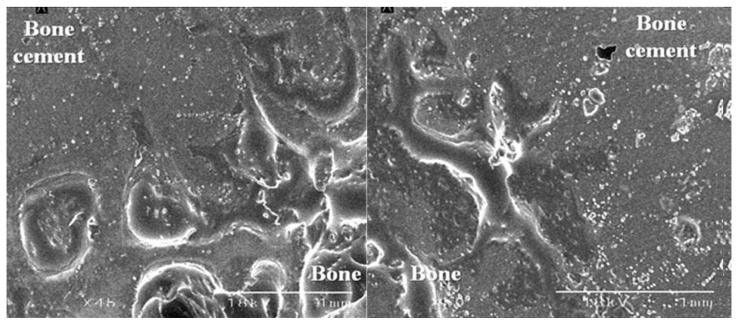
Scanning electron microscopy of the interface between the bone and bone cement. There was no gap between the bone and bone cement in all four pairs of the femoral specimens.

**Figure 8:**
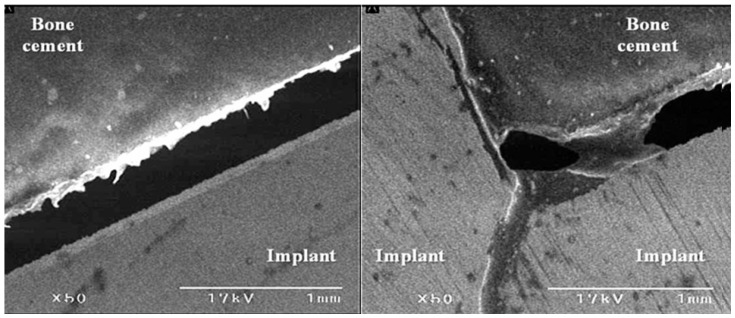
Scanning electron microscopy of the interface between the femoral head implant and bone cement.

With SEM, all the four pairs of the femoral specimens, obtained from both groups, showed no gap at the bone–cement interface (Figure 7). However, there was a gap of approximately 0.2 mm in size at the interface between the FHI and the bone cement (Figure 8).

## 4. Discussion

An appropriate experimental model of the human disease is a prerequisite for a clinical trial to assess the efficacy and safety of a novel treatment model in the setting of ONFH [1]. From this context, an animal model of ONFH played a key role in performing a pre-clinical trial to identify more effective treatments, whereas a cellular model was used to clarify the pathogenesis and pathophysiology of ONFH [50]. Nevertheless, many experimental models do not share the same physiological and metabolic characteristics with humans [51,52].

It is mandatory for orthopedic surgeons to obtain a complete understanding of human anatomy; anatomy is a basic medical discipline by which they can achieve improvements in their training. Moreover, cadaveric studies may allow orthopedic surgeons to study the characteristics of many diseases and anatomical structures that are vulnerable to damages, such as bone, muscle and ligament [53].

Cadaveric studies are also useful in performing an assessment of the biomechanics of anatomical structures, thus allowing orthopedic surgeons to develop new surgical techniques [53].

Biomechanical cadaveric studies can be performed when it is not easy to handle the movement or force of interest in the joint or soft tissue in vivo. This enables orthopedic surgeons to assess biomechanical characteristics and properties. Indeed, biomechanical cadaveric studies play a role in performing a pre-clinical assessment of new surgical techniques and implant designs [54,55,56]. This justifies the current biomechanical cadaveric study.

Mont MA et al. performed a systematic review of the previous published literature on untreated asymptomatic ONFH, thus showing that >25% involvement of the femoral head served as a risk factor of femoral head collapse [57]. Indeed, the HRA can be performed for patients aged < 50 years old who had necrotic lesions involving <30–33.3% of the femoral head [58,59].

To summarize, our results are as follows: First, changes in the degree of displacement at the final phase from the initial phase were calculated as 0.089 ± 0.036 mm in the experimental group and 0.083 ± 0.056 mm in the control group. However, this difference reached no statistical significance (*p* = 0.7789). Second, overall there was an increase in the degree of displacement due to the loading stress, with increased loading cycles in both groups. In cycles of up to 6000 times, there was a steep increase. After cycles of 8000 times, however, there was a gradual increase in it. Moreover, in cycles of up to 8000 times there was an increase in the difference in the degree of displacement due to the loading stress between the two groups. After cycles of 8000 times, however, such difference remained almost unchanged. Third, with SEM, all the four pairs of the femoral specimens, obtained from both groups, showed no gap at the bone–cement interface. However, there was a gap of approximately 0.2 mm in size at the interface between the FHI and bone cement.

However, our results cannot be generalized; further studies based on computational modeling are warranted to corroborate them. The necessity of computational modeling of musculoskeletal structures cannot be overlooked; it may be useful in not only providing the data about musculoskeletal structures, but also in simulating injuries and outcomes of surgical operations [60]. Thus, computational modeling and personalized simulations may provide fundamental insights into a better understanding of the pathophysiologic mechanisms underlying injuries. This can contribute to not only reducing the necessity of human or animal experiments, but also enabling orthopedic surgeons to implement novel treatment strategies or to make a plan for surgery [61]. To date, sophisticated approaches to the computational modeling of musculoskeletal structures have emerged. Indeed, such a model has been employed in studies about a specific type of implant or surgical procedure [62]. This is because a meticulous preoperative strategy based on computational modeling is of paramount importance when orthopedic surgeons choose the optimal type of implant [63].

The usefulness of computational modeling in the context of ONFH deserves special attention. It is more advantageous in predicting the whole process without actually performing the surgery as compared with a traditional static analysis [64].

Computational modeling with finite element analysis (FEA) plays a key role in the association with the design and development of a medical device [65]. More specifically, it can be used to assess the deformation field, strain field and stress field of the femoral head and support device [64]. It would, therefore, be worthwhile to explore the value of the FEA in the context of an MoM implant. Of note, the previous literature has simulated a loading by adopting a gait cycle reflecting the actual condition of an implant user [66,67,68,69,70].

An MoM implant is equipped with a higher stability and a lower risk of dislocations. It is harder as compared with ceramic materials; its advantages include a lower rate of fracture failure under high loads and a 20- to 100-fold lower rate of wear as compared with conventional metal-on-polyethylene implants [71]. Due to these benefits, an MoM implant may be used for younger and more active patients [72].

Efforts have been made to decrease the surface contact area (SCA) and to lower the rate of adhesion wear and the coefficient of friction [73]. A dimple is surface texturing that belongs to one such effort made for diverse types of mechanical components; it plays a role in trapping wear debris, preventing the abrasive wear of SCA and generating hydrodynamic pressure to provide additional lift [74,75]. Both theoretical and experimental studies have shown that surface texturing has a positive effect in improving the tribological performance of a device [76,77,78,79].

Jamari J. et al. assessed the effect of dimples on the rate of wear in the context of THA. These authors performed the FEA based on the prediction model with or without dimples. After simulations using 3D physiological loading of the joint under normal walking conditions, Jamari J. et al. showed that the dimples were effective in lowering the contact pressure and wear [80].

To date, orthopedic research has been driven by both biomechanical studies and clinical trials [81,82,83,84]. A cadaveric biomechanical study remains a useful method in that it allows surgeons, engineers and researchers to achieve results similar to in vivo clinical studies without endangering patients [53,85]. From this context, the current results are of significance in that this is a pilot experiment using a cadaveric model of the ONFH involving up to 50% of the remaining femoral head in Korea for future clinical studies. However, more efforts should be made to translate the current results into clinical practice.

## 5. Conclusions

In conclusion, our results indicate that orthopedic surgeons could consider performing the HRA in patients with ONFH where the bone defects involve up to 50% of the remaining femoral head without involving the femoral head–neck junction in the anterior and superior area of the femoral head. However, more evidence-based studies are warranted to justify our results.

## Figures and Tables

**Table 1:** The degree of displacement at each phase.

#	Values					
	Experimental Group (*n* = 10)			Control Group (*n* = 10)		
	Initial Phase	Final Phase	Δ	Initial Phase	Final Phase	Δ
1	0.237	0.450	0.213	0.051	0.121	0.07
2	0.032	0.066	0.034	0.078	0.190	0.112
3	0.069	0.151	0.082	0.055	0.140	0.085
4	0.031	0.061	0.03	0.109	0.240	0.131
5	0.123	0.251	0.128	0.025	0.073	0.048
6	0.098	0.199	0.01	0.140	0.308	0.168
7	0.042	0.090	0.048	0.075	0.146	0.071
8	0.077	0.172	0.095	0.045	0.104	0.059
9	0.088	0.201	0.113	0.068	0.162	0.094
10	0.062	0.143	0.081	0.052	0.103	0.051

Note: #, specimen identification number; Δ, changes in the degree of displacement at the final phase from the initial phase. All the values are presented at a unit of mm.

## Data Availability

All data generated or analysed during this study are included in this published article.
